# Identification and Molecular Analysis of Putative Self-Incompatibility Ribonuclease Alleles in an Extreme Polyploid Species, *Prunus laurocerasus* L.

**DOI:** 10.3389/fpls.2021.715414

**Published:** 2021-09-23

**Authors:** Júlia Halász, Anna Borbála Molnár, Gulce Ilhan, Sezai Ercisli, Attila Hegedűs

**Affiliations:** ^1^Group of Horticultural Plant Genetics, Department of Plant Biotechnology, Institute of Genetics and Biotechnology, Hungarian University of Agriculture and Life Sciences, Budapest, Hungary; ^2^Department of Horticulture, Faculty of Agriculture, Ataturk University, Erzurum, Turkey

**Keywords:** cherry laurel, self-incompatibility, *Prunus laurocerasus*, *S*-allele, *S*-genotyping, *S*-ribonuclease, trans-specific evolution

## Abstract

Cherry laurel (*Prunus laurocerasus* L.) is an extreme polyploid (2*n* = 22*x*) species of the Rosaceae family where gametophytic self-incompatibility (GSI) prevents inbreeding. This study was carried out to identify the *S*-ribonuclease alleles (*S*-RNases) of *P. laurocerasus* using PCR amplification of the first and second intron region of the *S-RNase* gene, cloning and sequencing. A total of 23 putative *S*-RNase alleles (*S*_1_–*S*_20_, *S*_5__m_, *S*_13__m_, and *S*_18__m_) were sequenced from the second (C2) to the fifth conserved region (C5), and they shared significant homology to other *Prunus S*-RNases. The length of the sequenced amplicons ranged from 505 to 1,544 bp, and similar sizes prevented the proper discrimination of some alleles based on PCR analysis. We have found three putatively non-functional alleles (*S*_5__m_, *S*_18__m_, and *S*_9_) coding for truncated proteins. Although firm conclusions cannot be drawn, our data seem to support that heteroallelic pollen cannot induce self-compatibility in this polyploid *Prunus* species. The identities in the deduced amino acid sequences between the *P. laurocerasus* and other *Prunus S*-RNases ranged between 44 and 100%, without a discontinuity gap separating the identity percentages of trans-specific and more distantly related alleles. The phylogenetic position, the identities in nucleotide sequences of the second intron and in deduced amino acid sequences found one or more trans-specific alleles for all but *S*_10_, *S*_14_, *S*_18_, and *S*_20_ cherry laurel RNases. The analysis of mutational frequencies in trans-specific allele pairs indicated the region RC4–C5 accepts the most amino acid replacements and hence it may contribute to allele-specificity. Our results form the basis of future studies to confirm the existence and function of the GSI system in this extreme polyploid species and the alleles identified will be also useful for phylogenetic studies of *Prunus S*-RNases as the number of *S-RNase* sequences was limited in the Racemose group of *Prunus* (where *P. laurocerasus* belongs to).

## Introduction

Gametophytic self-incompatibility (GSI) is a genetic mechanism that enables the plant to differentiate among pollen grains to be accepted or rejected for fertilization. Fertilization may only occur when the pollen *S*-allele is different from any of the *S*-alleles carried by the recipient pistil (McCubbin and Kao, 2000). The mechanism involves the interaction between pollen- and pistil-expressed proteins, both of which are encoded by a single polyallelic locus, the so-called *S*-locus. The protein accumulating in the stylar tissue is a ribonuclease enzyme called *S*-RNase (McClure et al., 1989), the pollen *S*-specificity is determined by an F-box containing protein (Tao and Iezzoni, 2010). The *S*-RNase is responsible for the rejection of the self-pollen by degrading the pollen RNA and consequently hindering the growth of the pollen tube (McClure et al., 1990). Mating selection in the *Prunus* genus (Rosaceae family) is known to operate by the GSI mechanism, and thus many economically significant fruit-bearing species exhibit self-incompatibility (SI) (Ashkani and Rees, 2016; Sassa, 2016).

*Prunus* has five subgenera including *Amygdalus*, *Cerasus*, *Prunus*, *Padus*, and *Laurocerasus* (Rehder, 1940). Chin et al. (2013, 2014) discerned three clades within *Prunus* genus that were named after their typical inflorescence structures. The “Solitary” clade included the peaches, almonds, apricots, and plums; the “Corymbose” group comprised cherry species; and the “Racemose” clade is composed of the deciduous *Padus* (e.g., *P. serotina* Ehrh. and *P. virginiana* L.) and the evergreen *Laurocerasus* (e.g., cherry laurel, *P. laurocerasus* L.) subgenera. Cherry laurel, a member of the ultimate group, is a docosaploid species possessing exceptionally high chromosome number (2*n* = 22*x* = 176) that exceeds those of other *Prunus* species (Meurman, 1929).

Polyploidy may affect the function of the GSI mechanism in various ways (Makovics-Zsohár and Halász, 2016). In the tetraploid Solanaceae and Malinae accessions, the pollen grains carrying two different *S*-alleles can grow in styles expressing the same *S*-alleles (Luu et al., 2001; Adachi et al., 2009; Qi et al., 2011), a phenomenon called competitive interaction. It is explained by the non-self-recognition model where the pollen *S*-proteins (SLF or SFBB) from each haplotype detoxify all but self *S*-RNases (Fujii et al., 2016). Hence, the presence of two different haplotypes in the heteroallelic pollen will have the capacity to detoxify all *S*-RNases. A similar phenomenon was first detected in a tetraploid *Prunus* species, *P. pseudocerasus* Lindl. cultivar (Huang et al., 2008), and later assumed in two additional cultivars of the species (Gu et al., 2013). However, the GSI mechanism in another tetraploid *Prunus* species, the sour cherry (*P*. *cerasus* L.) has been reported to operate in a different way, according to the “one-allele-match” model, in which the cause of self-compatibility (SC) could be traced back to the accumulation of non-functional *S*-haplotypes, i.e., mutated *S*-haplotypes that do not function properly (Hauck et al., 2006; Tao and Iezzoni, 2010). In a tetraploid accession, two such non-functional *S*-haplotypes must be present in the pollen to achieve self-fertilization. However, when the pollen grain contains at least one fully functional *S*-haplotype that has a match in the pistil, SI prevails. The analysis of the *S*-locus of hexaploid *P. domestica* L. has been initiated recently (Abdallah et al., 2019; Fernandez i Marti et al., 2021).

The pollination biology of cherry laurel was studied by Sulusoglu and Cavusoglu (2014b). Three genotypes had final fruit set ratios ranging from 0.0 to 5.7%, suggesting *P. laurocerasus* to be self-incompatible. Turkish experts and farmers also supported the SI phenotype of many cultivated varieties based on the observations that monovarietal orchards did not set fruit (Sulusoglu and Cavusoglu, 2014a; and personal communication). However, further analyses are required to test the (in)compatibility phenotype of more cultivars.

*Prunus* species are predominantly self-incompatible (Tao and Iezzoni, 2010; Hegedűs et al., 2012) and polyploidy in itself does not induce SC in such species (Hauck et al., 2006; Nunes et al., 2006). If the genetic background of the GSI system in *P. laurocerasus* is similar to Malinae species, a great number of *S*-haplotypes may result in mainly SC accessions. In contrast, if it works similar to *P. cerasus*, at least a total of 11 non-functional *S*-alleles should be accumulated in the genome to generate SC pollen, and accessions are likely to be SI. Although mutations may occur relatively frequently, since polyploidy suppresses purifying selection pressure as loss-of-function mutations in polyploid plants do not break the SI barrier immediately (Tsukamoto et al., 2006). But the genomic composition and the interaction or segregation of chromosomes are currently unknown in this species; and hence, it is hard to predict how the *S*-locus will work in *P. laurocerasus*. Apomixis may provide an opportunity to bypass the barrier of SI and form seed. Nonetheless, those seeds are formed without fertilization and hence with very different genetic consequences. The observation that most apomictic plants are polyploids suggests that polyploidy may trigger apomixis (Hojsgaard and Hörandl, 2019). However, from 200 *Prunus* species, 34% were polyploid without evidence available for a single apomictic species in this genus (Vamosi and Dickinson, 2006). Apomixis does not appear either in polyploid cherries (Dickinson, 2018) or plums like tetraploid blackthorn (Yeboah Gyan and Woodell, 1987; Guitián et al., 1993) and hexaploid European plums (Dickinson, 2018).

Cherry laurel is an ornamental and fruit-bearing shrub indigenous to the countries surrounding the Black Sea in the Balkan Peninsula, Western Asia and the Anatolian Peninsula (Ercisli, 2004; Sulusoglu, 2011). Multifarious varieties can be found mainly in northern Turkey in the so-called Black Sea Region where cherry laurel is widespread both in the natural vegetation and in cultivated areas and local gardens. Its fruit is a drupe that is commonly consumed as foodstuff in Turkey (Sulusoglu, 2011), and contains a huge amount of several bioactive compounds (Celik et al., 2011). The fruit has a significant role in the local folk remedy practices and recent studies have confirmed its several curative effects (Orhan and Akkol, 2011), suggesting the economic value of this species and its popularity in cultivation will steadily increase in future. Thus, studies are required to identify high-yielding varieties with the best commercial potential to be used in future breeding programs (Akbulut et al., 2007; Sulusoglu, 2011).

Cherry laurel as a docosaploid species in the Racemose group of *Prunus* genus, can be also used to clarify the GSI function in polyploid *Prunus* species. However, it requires careful analysis to initiate such studies as the high ploidy level may interfere with *S*-allele identification and *S*-genotype assignment. This study was carried out to identify and characterize the first putative *S*-*RNase* alleles in *P. laurocerasus* and evaluate the application and efficiency of *S*-genotyping assays. The molecular analysis of the alleles was aimed to screen for mutations putatively rendering the proteins non-functional, this information might be important both for breeding perspectives and in order to understand the function of *S*-locus in polyploid *Prunus*. The information obtained in this study is also used to increase our knowledge on the evolutionary relationships of *S-RNase* alleles within *Prunus*.

## Materials and Methods

### Plant Material

The leaf samples were collected from 50 phenotypically different cherry laurel (*Prunus laurocerasus* L.) accessions at the Black Sea region, Turkey; transported to the lab, and stored at –80°C.

### DNA Extraction and PCR Analysis for Initial Screening

Total genomic DNA was extracted from the leaf samples using the DNeasy Plant Mini Kit (Qiagen, Hilden, Germany) following the enclosed protocols. Part of the *S-RNase* gene – between the conserved regions C2 and C5, including the 2nd intron – was amplified by conducting PCR using the consensus primers PaConsII-F and PaConsII-R designed by Sonneveld et al. (2003). For the analysis of *S-RNase* gene first intron region, the PaConsI-F and PaConsI-R2 primers were used following the PCR temperature profile published by Sonneveld et al. (2006).

Approximately 40–60 ng of genomic DNA was used for PCR amplification in a 12.5 μL reaction volume, containing 10 × DreamTaq^TM^ Green buffer (Thermo Fisher Scientific, Waltham, MA, United States) with final concentrations of 1.5 mM MgCl_2_, 0.2 mM of dNTPs, 0.4 μM of the adequate primers, and 0.625 U of DreamTaq^TM^ DNA polymerase (Thermo Fisher Scientific). The PCR amplification was carried out in a 2720-type thermal cycler (Applied Biosystems, Foster City, CA, United States) in accordance with the protocol described for the primers (Sonneveld et al., 2003). The PCR products of 2nd intron regions were separated by electrophoresis on 1% TBE agarose gel after ethidium bromide (EtBr) staining at 80 V for 45 min and then the DNA bands were visualized by UV illumination. The approximate fragment sizes were determined by comparison to the GeneRuler^TM^ 1 kb DNA Ladder (Thermo Fisher Scientific). The evaluation of 1st intron region was based on the fluorescently labelled (FAM) forward primer and the amplicons were run on an automated capillary sequencer, ABI PRISM 3100 Genetic Analyzer (Applied Biosystems). ABI Peak Scanner 1.0 software and GS500 LIZ (Applied Biosystems) internal size standard were used for data analysis.

### Cloning and DNA Sequencing

The PCR products amplified by the PaConsII primer pair were chosen for cloning in case of ten cherry laurel genotypes, each having a unique pattern of differently sized bands. The most important aspects of the selection of samples used for sequencing were to identify as many alleles as possible and to check that fragments of the same size in different samples represent the same allele or not. Direct cloning of PCR products was carried out using pTZ57R/T vector (Thermo Fisher Scientific). The ligated plasmid vectors were transformed into JM109 *Escherichia coli* competent cells (Zymo Research Corp., Irvine, CA, United States). After the successfully transformed cells were visually selected by blue-white screening technique, the nucleotide sequences were determined for each fragment in both directions by using M13 sequencing primers. The differently sized plasmid DNA fragments were purified using the EZ-10 Spin Column Plasmid DNA kit (Bio Basic Inc., Markham, Canada) and then sequenced in an automated sequencer ABI PRISM 3100 Genetic Analyzer (Applied Biosystems).

### DNA Sequence Analysis

DNA and deduced amino acid (AA) sequences were compared using the NCBI BLASTN 2.11.0 software (Altschul et al., 1990) and the ClustalW program (Thompson et al., 1994) using the PAM (Percent Accepted Point Mutation) similarity matrix. The non-conservative amino acid replacements were defined as having negative scores in the matrix (Patthy, 2008). The aligned sequences were manually edited and presented using the BioEdit program v.7.2.5 (Hall, 1999). A phylogenetic tree was created with MEGA program v.7.0.26 (Kumar et al., 2016) using the “Minimum Evolution” method (Rzhetsky and Nei, 1992). The percentages of replicate trees in which the associated *S*-RNase allele sequences clustered together in the bootstrap test (1,000 replicates) were given next to the branches (Felsenstein, 1985). The tree is drawn to scale, with branch lengths in the same units as those of the evolutionary distances used to infer the phylogenetic tree. The evolutionary distances were computed using the JTT matrix-based method (Jones et al., 1992) and are in the units of the number of amino acid substitutions per site. The rate variation among sites was modeled with a gamma distribution (shape parameter = 0.8). The ME tree was searched using the Close-Neighbor-Interchange (CNI) algorithm (Nei and Kumar, 2000) at a search level of 1. The Neighbor-joining algorithm (Saitou and Nei, 1987) was used to generate the initial tree. The analysis involved 78 amino acid sequences. All positions with less than 95% site coverage were eliminated. That is, fewer than 5% alignment gaps, missing data, and ambiguous bases were allowed at any position. There was a total of 121 positions in the final dataset. Sequences of all the other *Prunus* species were obtained from the NCBI GenBank database.^1^

## Results

### *S*-Genotyping With Consensus Primers and Intron Polymorphism of the *Prunus laurocerasus S*-RNase

**FIGURE 1:**
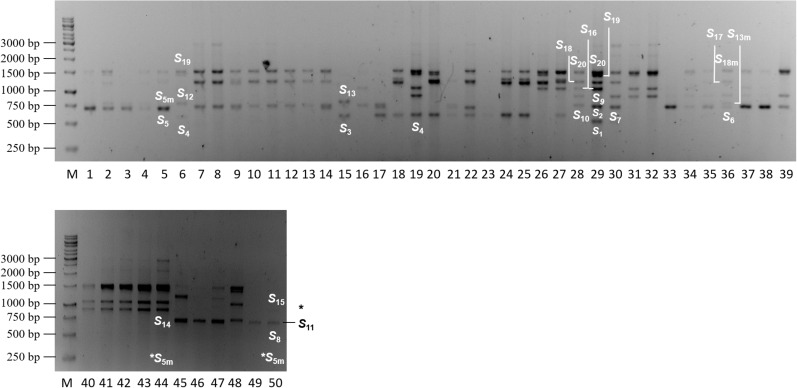
Agarose gel showing amplification products of PCR analysis conducted using the PaConsII-F and PaConsII-R consensus primers amplifying the second intron of the *Prunus laurocerasus S-RNase* gene. M: GeneRuler 1kb DNA Ladder (Thermo Fisher Scientific), 1–50: *Prunus laurocerasus* accessions; black asterisk indicates a faint but visible band, white asterisks show alleles determined by sequencing but not detected on the gel.

Since the second intron of the *Prunus S-RNase* is characterized by a considerable length polymorphism, the cherry laurel *S-RNase* alleles were first amplified using the consensus primers PaConsII-F and PaConsII-R designed by Sonneveld et al. (2003) for sweet cherry. The amplicons were separated on agarose gels. The PaConsII primers amplified the *S-RNase* gene in a total of 50 cherry laurel samples. The length of the amplicons ranged from approx. 500 to 3,000 bp and a maximum of seven bands were detected in a sample (Figure 1). It was remarkably lower than expected since owing to the high ploidy level of cherry laurel, a larger number of *S*-alleles was supposed to be amplified in each genotype. However, the thick and dense bands in several samples suggested the presence of more fragments with slightly different sizes.

The first intron of the *Prunus S-RNase* gene is smaller, and its length variations are also restricted compared to those of the second intron, but precise sizing on an automated DNA sequencer may provide information for *S*-genotyping. A subset of samples (1–23) was used to check if more alleles can be identified based on the first intron lengths. In this assay, 2–9 fragments/accession were detected, except sample 10 that did not amplify a single band. A total of 151 fragments were observed and assigned to 32 size categories, although some of those showed only 1-bp difference while other peaks had low intensity or showed traces of microsatellites, not allowing proper and reliable allele identification (Supplementary Table 1).

### Allele Identification Based on DNA Sequencing

Since *S*-PCR assays detected fewer alleles than expected from the ploidy level of cherry laurel (i.e., up to 22 bands per sample), we had to clarify whether only a limited number of alleles were carried by *P. laurocerasus* accessions or several alleles had matching intron lengths, resulting in a complex band in the agarose gel that contains fragments of different putative *S-RNase* alleles. Hence, DNA sequencing was carried out and amplicons of 10 *P. laurocerasus* accessions were cloned into vector and sent for DNA sequencing. Two putative *S-RNase* sequences were also determined in samples 44 and 50, although their corresponding bands were not observed in gel patterns.

In the ten samples, a total of 23 distinct nucleotide sequences were determined (one to five fragments per genotype). Using the newly obtained sequences as query, we performed a homology analysis with the BLASTN search tool in the NCBI GenBank database. The results provided statistical support (*E*-values ranged between 0.0 and 7e^–152^) for the homology with previously published *Prunus S-RNases*, confirming the novel sequences obtained from cherry laurel were indeed fragments of *S-RNases*. Each of the new *S*-alleles was assigned a number, starting from *S*_1_ to *S*_20_, ascending in the order of their increasing size, with three being a putative mutant version of others (*S*_5__m_, *S*_13__m_, and *S*_18__m_). All DNA sequences were submitted to the NCBI GenBank database under the accession numbers MG593769-MG593775, MG595258-MG595259, MG601104-MG601115, and MG922592-MG922593 (Table 1).

**TABLE 1:** The results of the BLASTN analysis of *Prunus laurocerasus* putative *S-RNase* alleles sequenced in this work, their corresponding NCBI nucleotide sequence database accession number, percentage of query coverage and identity, the *E*-value of alignment score and the closest homologs (species name, allele label, and accession number).

Allele	NCBI GenBank accession number	Coverage (%)	Identity (%)	*E*-value	The closest homolog (species and accession number)
*S*_1_	MG593769	98	97.8	0.0	*Prunus speciosa S*_22_-*RNase*, AB289876
*S*_2_	MG593770	98	97.1	0.0	*Prunus speciosa S*_34_-*RNase*, AB289887
		99	96.9	0.0	*Prunus cerasus S*_35_, EU054327
		94	97.1	0.0	*Prunus avium S*_27_, AY259112
*S*_3_	MG593771	99	99.7	0.0	*Prunus avium S*_6_, EU077236
		100	99.1	0.0	*Prunus avium S*_24_, AY259112
		100	98.1	0.0	*Prunus virginiana S*_4_, JF907560
*S*_4_	MG593772	99	97.5	0.0	*Prunus armeniaca S*_65_, JQ327151
		99	97.3	0.0	*Prunus armeniaca S*_48_, HQ342878
		100	97.0	0.0	*Prunus armeniaca S*_24_, HQ615602
		98	97.0	0.0	*Prunus armeniaca S*_38_, GU586228
		95	97.9	0.0	*Prunus tenella S*_1_, DQ983373
		100	95.8	0.0	*Prunus armeniaca S*_4_, AY587564
		91	98.0	0.0	*Prunus mira S*_1_, AB597200
		88	94.8	0.0	*Prunus virginiana S*_2_, JF907558
*S*_5_	MG593773	98	93.8	0.0	*Prunus virginiana S*_5_, JQ627793
		98	87.1	0.0	*Prunus domestica S*_*f*_, MW407938
*S*_5__m_	MG922592	98	93.5	0.0	*Prunus virginiana S*_5_, JQ627793
*S*_6_	MG593774	99	94.0	0.0	*Prunus spinosa S*_3–1_, DQ677584
		100	93.2	0.0	*Prunus laurocerasus S*_8_, MG595258
		90	94.7	0.0	*Prunus virginiana S*_6_, JF907562
		83	96.7	0.0	*Prunus armeniaca S*_17_, EU516388
		47	97.6	8e-158	*Prunus mume S*_11_, EU020118
		57	97.0	2e-154	*Prunus mume S*_15_, EU020122
*S*_7_	MG593775	98	93.1	0.0	*Prunus dulcis S*_55_, FN599511
		99	93.0	0.0	*Prunus dulcis S*_40_, HQ622703
		99	92.6	0.0	*Prunus armeniaca S*_66_, JQ327152
		98	92.0	0.0	*Prunus webbii S*_k_, AM690360
*S*_8_	MG595258	99	98.7	0.0	*Prunus spinosa S*_3–1_, DQ677584
		100	93.2	0.0	*Prunus laurocerasus S*_6_, MG593774
		89	93.8	0.0	*Prunus virginiana S*_6_, JF907562
		83	98.0	0.0	*Prunus armeniaca S*_17_, EU516388
*S*_9_	MG595259	94	96.8	0.0	*Prunus avium S*_13_, DQ385842
		56	98.5	8e-163	*Prunus mume S*_11_, EU020118
		56	97.9	2e-159	*Prunus mume S*_15_, EU020122
		57	99.4	2e-173	*Prunus tenella S*_17_, KU167069
*S*_10_	MG601104	87	93.2	0.0	*Prunus dulcis S*_54_, AY613341
		87	93.7	0.0	*Prunus dulcis S*_63_, AY613919
		87	93.7	0.0	*Prunus dulcis S*_56_, AY613343
		85	93.4	0.0	*Prunus armeniaca S*_49_, HQ342879
		86	92.9	0.0	*Prunus armeniaca S*_50_, HQ342880
		88	96.8	0.0	*Prunus virginiana S*_7_, JQ627795
*S*_11_	MG601105	99	98.4	0.0	*Prunus speciosa S*_3_, AB289860
		99	97.5	0.0	*Prunus virginiana S*_6_, JF907562
		96	97.0	0.0	*Prunus armeniaca S*_40_, GU354239
*S*_12_	MG601106	99	94.1	0.0	*Prunus speciosa S*_44_, AB289895
		99	93.1	0.0	*Prunus simonii S*_1_, EU376959
		95	93.1	0.0	*Prunus salicina S*_h_, AB084148
*S*_13_	MG601107	93	99.7	0.0	*Prunus avium S*_9_, AJ635270
*S*_13__m_	MG601108	93	93.2	0.0	*Prunus avium S*_9_, AJ635270
*S*_14_	MG601109	92	96.3	2e-175	*Prunus dulcis S*_9_, MH316092
*S*_15_	MG601110	100	93.8	0.0	*Prunus spinosa S*_7_, EU833958
*S*_16_	MG601111	70	93.4	2e-163	*Prunus japonica S*_1_, EF635417
*S*_17_	MG601112	44	94.0	0.0	*Prunus serotina S*_3_, MN098835
*S*_18_	MG601113	75	84.9	0.0	*Prunus armeniaca S*_C_, DQ422947
*S*_18__m_	MG922593	65	85.0	0.0	*Prunus armeniaca S*_C_, DQ422947
*S*_19_	MG601114	77	92.5	0.0	*Prunus tenella S*_9_, DQ983370
		62	92.3	0.0	*Prunus armeniaca S*_25_, EU037264
		76	94.1	0.0	*Prunus dulcis S*_54_, FN599510
		29	98.0	5e-168	*Prunus dulcis S*_n_, DQ093825
*S*_20_	MG601115	36	88.9	7e-152	*Prunus armeniaca S*_1_, AY587561

**FIGURE 2:**
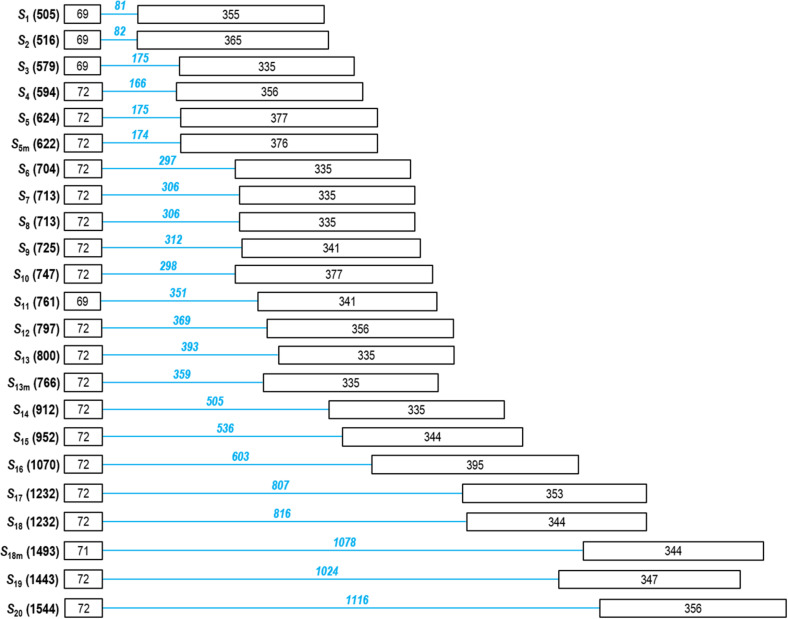
Structure of the partial *Prunus laurocerasus* putative *S-RNase* alleles. Boxes and lines represent exons and introns, respectively (not to scale). The DNA sequence was determined between the C2 and C5 conserved regions, and the lengths of exon 2 and 3 (numbers in black) as well as the second intron (numbers in blue) are shown.

DNA sequencing confirmed the presence of complex bands. In sample 5, the band under 750 bp contained *S*_5_ and *S*_5__m_; in sample 29, the band over 500 bp contained *S*_1_ and *S*_2_; and the band at 1,500 bp in the same sample contained both *S*_19_ and *S*_20_. In addition, the discrimination of the *S-RNase* alleles, *S*_3_/*S*_4_; *S*_6/_*S*_7_/*S*_8_/*S*_9_/*S*_10_/*S*_11_; *S*_12_/*S*_13_; *S*_14_/*S*_15_; *S*_17_/*S*_18_; and *S*_18__m_/*S*_20_ is a challenging task as their bands were positioned into nearly identical regions of the agarose gel. DNA sequencing indicated that size variation among those practically indistinguishable fragments ranged between 0 (*S*_17_ and *S*_18_) and 57 bp (*S*_6_ and *S*_11_) (Figure 2). For these reasons, we only assigned allele labels to the sequenced and reliably identified fragments shown in Figure 1.

### Molecular Analysis of *Prunus laurocerasus S-RNase* Alleles

**FIGURE 3:**
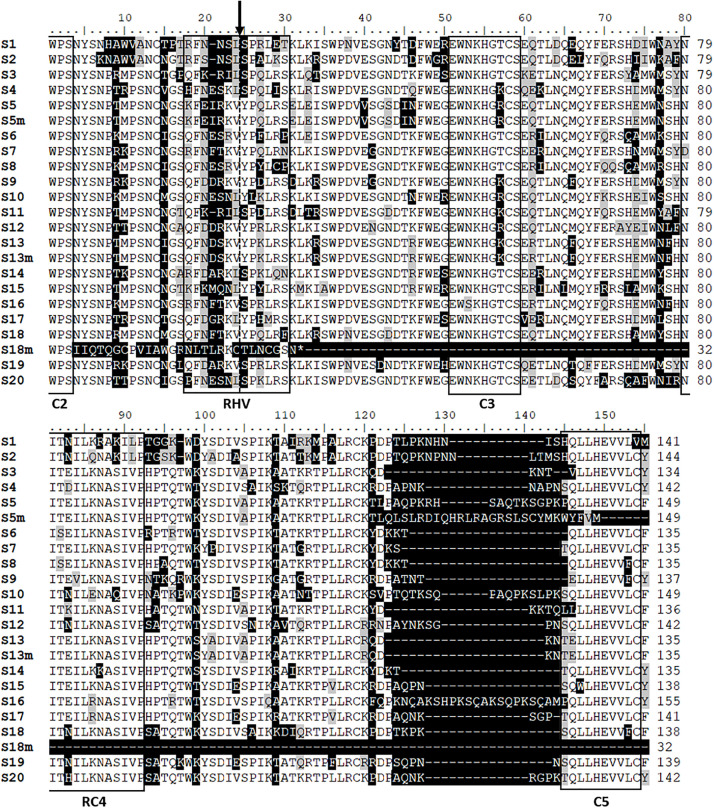
Alignment of deduced partial amino acid sequences of the newly identified *Prunus laurocerasus* putative *S*-RNase alleles, *S*_1_–*S*_20_, and the three mutant alleles (*S*_5__m_, *S*_13__m_, and *S*_18__m_). The conserved (C2, C3, RC4, and C5) and the hypervariable region (RHV), characteristic of rosaceous *S*-RNases are boxed according to Ushijima et al. (1998). The position of the 2nd intron is indicated by an arrow. Partially conserved and highly polymorphic residues are shown against gray and black background colors, respectively.

The binding sites of the primers defined the length of amplified region that covered most of the *Prunus S-RNase* gene, including the coding region from C2 to C5 conserved motifs and the 2nd intron. The lengths of the sequenced fragments ranged between 505 and 1,544 bp, the size polymorphism was mainly due to the differences in the 2nd intron length (81–1,116 bp). The shortest deduced AA sequence of the *S-RNase* alleles was composed of 32 AA residues that is due to a premature stop codon. All other sequences are predicted to contain 134–155 AA residues, the difference is mainly attributed to the indel region upstream of C5, although some single-nucleotide gaps were also observed within the RHV region and just downstream of RC4. The alignment of the deduced *S*-RNase AA sequences of the *S*_1_–*S*_20_, *S*_5__m_, *S*_13__m_, and *S*_18__m_ alleles is shown in Figure 3.

Pairwise comparisons revealed that the nucleotide sequences of exons had an average similarity of 80.0% ranging from 64.0% (*S*_2_- and *S*_5_-alleles) to 97.0% (*S*_6_- and *S*_8_-alleles); whereas the deduced AA sequences of the *S-*RNases had an average identity of 69.1%, (Supplementary Table 2). The *S*_2_-*S*_5_ and *S*_6_-*S*_8_ allele pairs showed the lowest (52.9%) and highest (93.3%) sequence identities in their AA sequences, respectively.

### Characterization of the Mutated Cherry Laurel *S-RNases*

Sequence variations were found in case of three newly identified *P. laurocerasus S-RNase* alleles (*S*_5_, *S*_13_, and *S*_18_), and we labeled them as *S*_5__m_, *S*_13__m_, and *S*_18__m_. The similarities between the wild-type and mutated alleles were 99.3, 99.2, and 97.8% for the allele pairs *S*_5_/*S*_5__m_, *S*_13_/*S*_13__m_, and *S*_18_/*S*_18__m_, respectively. The nucleotide sequences of such *S*-allele pairs were rather similar with just a limited number of alterations due to putative mutations, mainly involving SNPs and indels (Supplementary Figure 1). The deduced AA sequences of the *S*_13_ and *S*_13__m_ alleles were identical (Figure 3). Most of the differences, such as a 35-bp deletion, four microindels, and 10 SNPs, occurred within the 2nd intron region (Supplementary Figure 1B). In the coding region of the *S-RNase* gene, there were only three synonymous substitutions, which suggests the functional identity of the wild-type and mutated alleles.

Besides sequence alterations in the 2nd intron, the coding region of the *S*_5__m_ and *S*_18__m_-*RNases* also carried mutations, some of which could be of serious consequence to the structure and function of the encoded proteins. In case of the *S*_5__m_*-RNase*, a single cytosine was deleted in the 3rd exon of the gene that results in a frame-shift mutation just upstream of the C5 region (Supplementary Figure 1A) and hence the protein is likely to lack this conserved region (Figure 3). Another frame-shift mutation was detected in the *S*_18__m_*-RNase* nucleotide sequence due to the loss of an adenine nucleotide (Supplementary Figure 1C). This mutation resulted in an abnormal amino acid sequence downstream of C2 that leads to a premature stop codon. Hence, this allele, if transcribed, encodes a putatively truncated protein lacking a major part of the RNase enzyme including the RHV, C3, RC4, and C5 regions (Figure 3). Other sequence alterations occurred frequently in both the intron and exon regions of this allele. The *P. laurocerasus S*_9_-*RNase* allele carries an A-to-G change at the penultimate nucleotide position of the 3’ splice site of the second intron, that may affect intron splicing efficiency.

### Phylogenetic Analysis and Evidence for Trans-Specific Evolution

The newly identified cherry laurel *S-RNase* sequences were clarified to be homologous to other *Prunus S-RNases* (Table 1). Since identities between the pairs of deduced AA sequences were always lower than those of nucleotide sequences (Supplementary Table 2), we performed a BLASTP search in the NCBI protein database using the deduced AA sequence of cherry laurel *S*-ribonuclease as a query. The *E*-values confirmed homology to *Prunus S*-RNases, and the identity ratios ranged between 81 and 100% (Supplementary Table 3). Some *P. laurocerasus S*-RNase proteins (*S*_2_, *S*_3_, *S*_4_, *S*_5_, *S*_7_, *S*_8_, *S*_9_, *S*_11_, *S*_12_, *S*_13_, *S*_17_, and *S*_19_) showed identities over 96% to one or more database sequences, others (*S*_1_, *S*_6_, *S*_10_, and *S*_15_) showed identities slightly lower than 96%, while the remaining alleles (*S*_14_, *S*_16_, *S*_18_, and *S*_20_) were characterized by smaller identities to database sequences, ranging from 80 to 94%. Though the second intron of the same *S-RNase* alleles showed more pronounced divergence, some of them were also highly similar (their identities ranged from 17.9 to 99.8%).

Considerable identities were found between some of the newly identified cherry laurel (*Plau*) *S*-alleles and their closest homologs. The amino acid sequences of *S*_3_ and *S*_13_ were identical to sweet cherry (*Pav*) *S*_6_ and *S*_9_-RNases, respectively, with the only difference being a single nucleotide substitution in the second intron region in both cases. However, the sequence of *Pav-S*_9_ available in the database was considerably shorter than *Plau-S*_13_ determined by us. Besides *Pav*-*S*_6_, *Plau*-*S*_3_ was also similar to *Pav*-*S*_24_ and *Pvirg*-*S*_4_. The two sweet cherry alleles were reported to be different only in the C1–C2 region (Wünsch and Hormaza, 2004). Interestingly, *Plau*-*S*_3_ is identical to both alleles in the C2–C5 region. Their introns are also nearly identical with a single base difference. The *Pvirg*-*S*_4_ had three amino acid replacements, a 4-bp insertion and a single base substitution in the second intron compared to other alleles in this group. It supports their common evolutionary origin.

The *Plau*-*S*_9_-RNase shared 99.2% identity with *Pav-S*_13_, with a single conservative amino acid replacement. The *Plau*-*S*_11_-RNase showed 98.5% identity with a *P. speciosa* (Koidz.) Ingram (*Pspec*) *S*_3_-RNase both in the deduced amino acid and second intron sequences. Apricot (*Parm*) *S*_40_-RNase was somewhat less similar to *Plau*-*S*_11_. *Plau*-*S*_1_ and *Pspec-S*_22_ differed in only four amino acids and two nucleotides in the 2nd intron confirming their close relationship.

The *Plau*-*S*_4_-RNase showed identities over 98% to 5 *P. armeniaca* L. alleles (*Parm*-*S*_4_, *S*_24_, *S*_38_, *S*_48_, and *S*_65_), while their second intron sequence shared identities over 90%. The allele-specificity of *Parm*-*S*_24_, *S*_38_, and *S*_48_ should be checked as they have identical 2nd intron sequences and almost identical AA sequences. *P. tenella* (*Pten*) *S*_1_ and especially *P. mira* (*Pmir*) *S*_1_ were the most similar to *Plau-S*_4_. They were also very similar to the AA sequence of *Parm*-*S*_4_ and *S*_65_, although their introns showed more differences. The *P. virginiana* (*Pvirg*) *S*_2_ shared 94% identity with *Plau*-*S*_4_ in their deduced amino acid sequences while their introns were rather dissimilar (29% identity). At least some *P. armeniaca*, the *P. tenella* and *P. mira S*_1_-alleles might be trans-specific as their AA and intron sequences were more than 96% identical. However, the *Pvirg*-*S*_2_ is similar but seems not to belong to this trans-specific group, what is further supported by the fact that two AA replacements were evident in the RHV region.

The *Plau*-*S*_2_-, *S*_5_-, *S*_7_-, *S*_12_-, *S*_15_-, *S*_17_-, and *S*_19_-alleles showed considerable identities in their deduced AA sequences to their closest homologs while their introns were more disparate, with identities ranging between 20 and 94%. *Plau*-*S*_2_ and *Pav*-*S*_27_ showed only 31% identity in their 2nd intron sequences due to a 93-bp insertion and several SNPs. The sweet cherry allele also differs from *Pspec*-*S*_34_ and *Pcer*-*S*_35_, while *Plau*-*S*_2_ is in a more distant phylogenetic position. *Plau*-*S*_10_ had 95.9% identity to *Pvirg*-*S*_7_, exceeding the similarity shared with some *P. armeniaca* and *P. dulcis* (Mill.) D. A. Webb (*Pdul*) alleles. Their introns were rather different. The *Plau*-*S*_17_ was also found to be 99.2% identical to the *P. serotina* (*Pser*) *S*_3_ allele; however, the comparison was made based on a shorter alignment and the introns were rather dissimilar. The *Plau*-*S*_6_ and *Plau*-*S*_8_-RNases shared 93 and 87% similarities in AA and intron sequences, respectively, and were also similar to specific *P. virginiana*, *P. spinosa* L., *P. mume* (Sieb.) Sieb. and Zucc., and *P. armeniaca S*-RNases. Within this clade, *Plau*-*S*_8_ and *P. spinosa* (*Pspi*) *S*_3__–__1_ were the most similar sequences supporting their relationships, while *Pvirg*-*S*_6_ was nearly at an equal distance from both *Plau*-*S*_6_ and *S*_8_, and mainly their introns differed with *Pvirg*-*S*_6_ having many indels.

**FIGURE 4:**
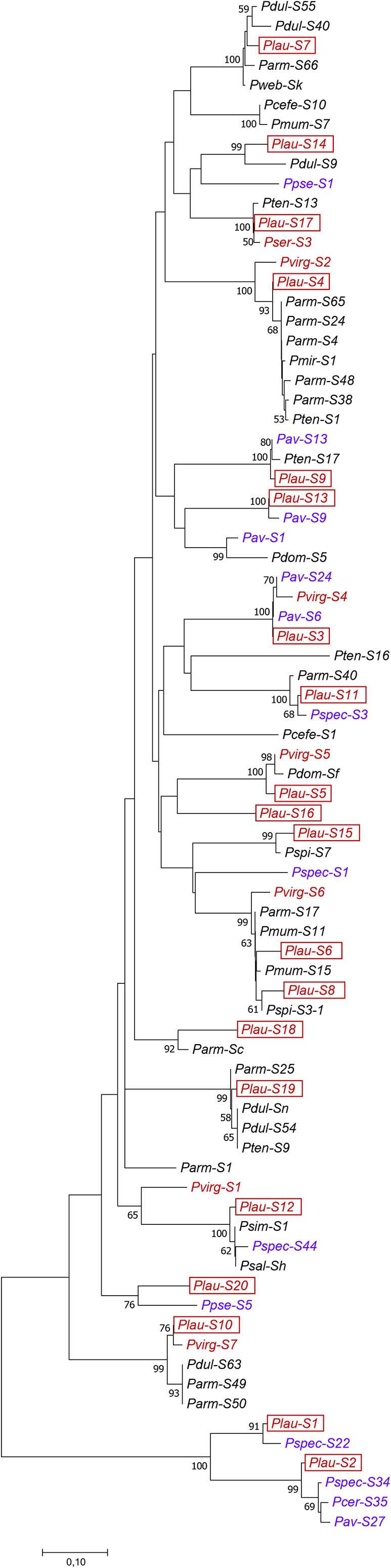
Phylogenetic tree of the new *P. laurocerasus* and other *Prunus S*-RNase sequences constructed using the Minimum Evolution method with the indication of the bootstrap values (%, *n* = 1,000). Black, blue, and maroon colors represent distinct groups of Solitary, Corymbose, and Racemose groups within *Prunus*, respectively. The labels of novel cherry laurel *S*-alleles are framed. The accession numbers of the sequences used in the analysis can be found in Supplementary Table 3.

The pairs of the *Plau*-*S*_14_, *S*_18_, and *S*_20_-RNase alleles and their respective closest homologs showed identities of 80–90% in their deduced AA sequences and their 2nd introns were also considerably dissimilar (18–63%), arguing against their trans-specific origin, which was also confirmed by their position in the phylogenetic tree (e.g., *Plau*-*S*_20_ and *Parm*-*S*_1_) (Figure 4). Those alleles share more distant evolutionary relationships. The *Plau*-*S*_16_-RNase allele and its closest homolog in the database (*P. japonica S*_1_) requires further analysis as their introns matched in 64% of the nucleotides and the deduced AA sequence of *P. japonica S*_1_-allele was only available between C2–C3, allowing the comparison of the alleles only on a shorter region.

The evolutionary history of *P. laurocerasus* and other *Prunus S*-RNases (including the most similar database sequences to each of the identified cherry laurel alleles and some arbitrarily chosen sequences that were available from C2 to C5) was inferred from an alignment of the deduced AA sequences and using the Minimum Evolution method (Figure 4). The optimal tree with the sum of branch length equals to 5.22 is shown with bootstrap values higher than 50%. *P. laurocerasus* sequences clustered with *S*-RNase alleles of other species and the most clades received significant bootstrap support. The similarities among sequences in the alignment ranged from 44.4 (*Pav*-*S*_9_ and *Pcer*-*S*_35_) to 100% (*Plau*-*S*_3_ and *Pav*-*S*_6_; *Pmum*-*S*_11_ and *Parm*-*S*_17_; *Psal*-*S*_h_ and *P. simonii* Carr. *S*_1_, and *Pdul*-*S*_n_ and *Pten*-*S*_9_) (Supplementary Table 4). The phylogenetic tree clustered the cherry laurel *S*-RNases with their closest homologs according to their shared similarity, while greater branch lengths indicated the evolutionary distances between four alleles (*S*_10_, *S*_14_, *S*_18_, and *S*_20_) and their closest homologs having lower identities in both their deduced AA and intron sequences. The *Plau*-*S*_20_ allele did not cluster with its closest homolog (Figure 4). For *S*_16_ and *S*_17_, the AA sequences of their database homologs were shorter and hence their phylogenetic relationships cannot be confirmed or ruled out.

The *Plau-S*_5_, *S*_10_, and *S*_17_-RNase sequences positioned closer to sequences in the “Racemose” group than to the sequences in the “Solitary” group. In contrast, the *Plau*-*S*_4_ was closer to apricot (belonging to the “Solitary” group) sequences than the *Pvirg*-*S*_2_. The *S*_9_ and *S*_11_-alleles clustered with sequences both from the “Corymbose” and “Solitary” groups and in those clades *P. laurocerasus* sequences were closer to the “Corymbose” than the “Solitary” alleles.

**FIGURE 5:**
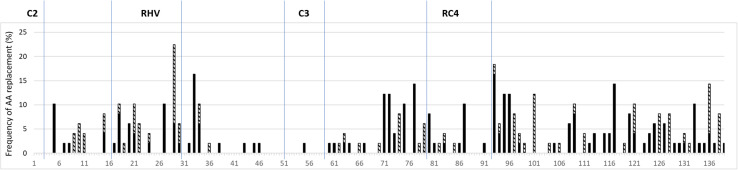
Frequency of mutations resulting in conservative (black columns) and non-conservative (hatched columns) amino acid replacements in different regions of the *Prunus S*-ribonuclease gene. The numbers in axis x refer to the amino acid positions in the alignment, and conserved and RHV regions of the protein are labeled according to Ushijima et al. (1998).

A total of 237 mutations were detected among the 55 pairs of sequences analysed in this study, 33% of which was non-conservative AA replacements. A limited number of AA replacements was identified in the conserved regions C3 and RC4 (C2 and C5 were not studied as only partial sequences were available) of the *S*-RNase protein, most of which occurred in the RC4. The RHV contained 16% of all the mutations. The regions between known structural motifs, C2-RHV, RHV-C3, C3-RC4, and RC4-C5 accumulated 8, 8, 17, and 43% of the total amount of mutations. The amino acid replacements and the non-conservative replacements were the most frequent in the RC4-C5, C3-RC4, and RHV regions (Figure 5, the original data can be seen in Supplementary Table 5).

## Discussion

### Allele Number and Genotyping Efficiency

The size of amplicons including the 2nd intron region of the *S-RNase* gene ranged from 500 to 3,000 bp, and its polymorphism was mainly attributable to intron size variations. The 2nd intron of *Prunus S-RNase* alleles has been long known to be highly polymorphic in its length (Tao et al., 1999; Tamura et al., 2000); and it could be also used for the *S*-genotyping of *P. laurocerasus*. Similar size ranges were detected in other species, such as almond (80–2,872 bp, Ortega et al., 2006), apricot (500–2,800 bp, Halász et al., 2007), and sweet cherry (577–2,385 bp, Sonneveld et al., 2003).

We detected a relatively limited number of fragments compared to the polyploid genome of *P. laurocerasus*. Fewer than expected alleles in samples can be attributed to several reasons. Preferential amplification was reported to be a major contributor to the failure of the amplification of certain alleles relative to others (Walsh et al., 1992). Also, a polyploid plant can have multiple genomic copies of a single allele that might be observed as a thick, intense band on the agarose gel. All such factors can considerably decrease the number of detected fragments. Furthermore, it is hard to identify a distinct allele when two or more fragments are nearly equal in their lengths and thus cannot be separated from each other on the agarose gel (Sonneveld et al., 2003; Nantongo et al., 2016).

Although the first two options could not be checked, DNA sequencing of the amplicons confirmed the presence of fragments representing different *S*-alleles and having almost identical or very similar sizes. The gel was run for long time to allow separation but size differences under 60 bp could not be reliably resolved, not to speak about the alleles *S*_17_ and *S*_18_ that were identical in size (1,232 bp) while their nucleotide and deduced AA sequences showed considerable differences with only 82.4 and 72.3% pairwise identities, respectively. Fragments with seemingly identical sizes are the most frequently considered to represent a single allele, a strategy that is reliably used in diploid *Prunus* species. However, some difficulties were also reported in diploid species (Sonneveld et al., 2003; Ortega et al., 2005; Halász et al., 2007), our results confirmed that the most *S-RNase* alleles cannot be identified and discriminated based on intron length polymorphism in an extreme polyploid species. The size of the first intron region was known to be less variable compared to 2nd intron, what has been also observed in *P. laurocerasus S-RNase* alleles. In conclusion, the reliable identification of *S*-alleles requires DNA sequencing or other assays (e.g., allele-specific PCR or digestion with allele-specific restriction endonucleases, segregation analysis).

The seemingly large number of 23 distinct putative *S-RNases* in cherry laurel is not unprecedented, since up to now more than 30 *S*-alleles have been identified in several other *Prunus* species, including apricot, almond, or sweet cherry (Cachi et al., 2017; Herrera et al., 2018; Gómez et al., 2019). In contrast, peach, a generally SC species was reported to have only four *S*-alleles (Hanada et al., 2014). The *S*-allele polymorphism is crucially important for a SI species to increase the chance of mating (Schueler et al., 2006). Therefore, the number of *S*-alleles may suggest that a functional GSI system operates in cherry laurel.

### Putatively Non-functional *S*-*RNases*

Three pairs of alleles showed only minor differences and we labeled those alleles with the same number as their non-mutated pair and the letter “m,” indicating it is a stylar-part mutant. Two of the three mutated alleles (*S*_5__m_ and *S*_18__m_-*RNases*) were found to contain a single nucleotide deletion in the coding region of the gene, which is likely to induce a frame-shift mutation and the formation of a premature termination codon. The deduced amino acid sequence of the *S*_5__m_*-RNase* lacked the C5 conserved region. The replacement of a cysteine residue in the peach *S*_2__m_-*RNase* by tyrosine prevented the accumulation of the RNase protein in the stylar tissue and resulted in the loss of ribonuclease activity (Tao et al., 2007). It confirms the C5 region is important for the structural stability and enzyme activity of RNase proteins. The *P. salicina* Lindl. *S*_*C*_-RNase (ABG36936) allele was found to have a similar microdeletion upstream of C5, although no information was available for its function. Since *P. salicina* is a diploid species, the phenotypic consequences of such mutations might be easily checked. The *S*_18__m_*-RNase* putatively lacks a major part of the coding regions resulting in the loss of RHV to C5 regions. Since those regions are involved in enzyme catalysis, the formation of protein fold and allele-specificity (Ioerger et al., 1991; Ushijima et al., 1998), this protein is unlikely to be functional.

Another mutation was found in the *P. laurocerasus S*_9_-*RNase* allele that carries an alteration in the 3’ splice site of 2nd intron. An A-to-G substitution at the penultimate position of the intron changes the AG canonical 3’ splice site to GG. The AG dinucleotide is highly conserved at the acceptor splice site, and all such changes described previously resulted in intron retention or the activation of the nearby cryptic 3’ splice sites both in human and plant genes (McNellis et al., 1994; Harteveld et al., 1996). The unspliced mRNA of the *S*_9_-*RNase* will lead to the addition of 13 novel amino acids before a new stop codon (TAA) is reached. Such a truncated protein would be surely non-functional because of the shortage of important structural regions. The possible activation of all putative cryptic splicing sites would also result in stop codons and the formation of truncated proteins that might be checked by cDNA sequencing.

We have identified three putatively non-functional alleles, and further analyses are likely to detect more. The *S*-locus mutations inducing SC in *Prunus* more frequently affect the pollen *S-haplotype-specific F-box* (*SFB*) gene than the stylar expressed *S-RNases* (Hegedűs et al., 2012; Socias et al., 2015). A detailed study (cDNA sequencing and identification of *SFB*-alleles) may help finding additional SC haplotypes. If similarly to *P. cerasus*, the one-allele-match model was responsible for the breakdown of GSI (Hauck et al., 2006), only the accessions having a minimum of eleven non-functional *S*-haplotypes would be SC in this docosaploid species, presenting a small chance of being self-compatible. An alternative hypothesis involves that SC is induced by the heteroallelic pollen, similarly to what was hypothesized in *P. pseudocerasus* but to this day it has not yet been validated (Huang et al., 2008; Gu et al., 2013). If segregation were disomic, the increasing chromosome number would result in the increase of chance to be SC. Considering the number of *S*-alleles detected in this study, the formation of heteroallelic and hence SC pollen should be frequent. However, the *S*-allele number detected in the present study and published data support *P. laurocerasus* is self-incompatible (Sulusoglu and Cavusoglu, 2014a,b), what suggests SC is most likely achieved according to the one-allele-match model in *P. laurocerasus*, similarly to *P. cerasus* (Hauck et al., 2006). In addition, another tetraploid species, *P. spinosa* was also shown to carry four different *S*-haplotypes while being SI (Nunes et al., 2006). The heteroallelic pollen induced SC is only consistent with the non-self-recognition model, while in *Prunus*, a self-recognition mechanism has been described (Fujii et al., 2016). However, new experiments are needed to confirm that the low fruit set after self-pollination is not explained by other phenomena (e.g., inbreeding depression) (Ortega et al., 2010) and the *S*-genotyped accessions are in fact SI. Hence, our results do not allow to make firm conclusions on the genetic model controlling the breakdown of SI in this polyploid species but provide the early information that will be useful for future clarification.

### The Evolution of *S-RNase* Alleles in *Prunus*

The identities among nucleotide sequences were higher than those among the deduced amino acid sequences, similarly to studies in other *Prunus S*-RNases (Gu et al., 2012). The *S-*RNase sequence identities observed in cherry laurel were very similar to those of almond *S*-RNases, varying from 46.6 to 93.9% at the amino acid level (Ortega et al., 2006). However, similarly to former studies we could also find some interspecific identities exceeding the intraspecific identities between *P. laurocerasus S*-RNases (52.9–93.3%) and reaching 100% identity in two cases (Supplementary Table 3). For 12 *P. laurocerasus S*-RNases we have found one or more homologs sharing identities over 96%, 4 others had slightly lower identities to their closest homologs and 4 *P. laurocerasus S*-RNase alleles had only homologs with markedly lower identities (81–93%).

The higher inter- than intraspecific identities among *S*-RNase alleles can be explained by their probable common origin preceding the separation of the evolutionary lineages of studied species. This pattern is called trans-specific evolution and has been described many times for *S*-RNase alleles (Ioerger et al., 1990; Ushijima et al., 1998; Igic and Kohn, 2001; Sutherland et al., 2008). Right after the speciation event, however, the trans-specific alleles start to evolve separately from each other in sexually isolated descendant species. This implies that the earlier the two sequences separated, the more time they had to accumulate independent mutations, and hence, the more they would differ. Alternatively, the fact that two separate species share a common *S*-RNase may also be explained by introgressive hybridization. However, it is not feasible for *P. laurocerasus* since crosses between a docosaploid and diploid or tetraploid species (e.g., *P. avium* L., *P. speciosa*) would result in the dramatic change of ploidy levels.

Sutherland et al. (2008) identified six pairs of trans-specific *Prunus S*-RNases and detected a large discontinuity of 11% separating the AA identity ranges of trans-specific (96–100%) and other alleles (71–85%). The sequence identity matrix obtained from the alignment of 20 *P. laurocerasus* and 58 other *S*-RNase alleles declared the identities ranged between 44 and 100%, and the whole range was evenly covered by pairwise identity percentage values. It suggests the identification of a great number of *Prunus S*-RNases since 2008 (and especially from the less studied Racemose group) can help to have a more nuanced and reliable outcome of evolutionary inferences. The evolution of *S*-RNase alleles is an ongoing process and the more alleles we identify make the picture more complex. Nonetheless, the loss of a discontinuity gap in identity percentages between the trans-specific and other alleles also makes the recognition of trans-specific alleles more complicated.

We used a multi-approach analysis encompassing phylogenetic studies and the determination of identity percentages among deduced amino acid and nucleotide sequences of the *S-RNase* gene and its 2nd intron, respectively. For *Plau*-*S*_1_-*S*_9_, *S*_11_-*S*_13_, *S*_15_, *S*_17_, and *S*_19_, data indicate they are trans-specific to one or all their closest homologs found in the database. In general, introns were more different than the deduced AA sequences. The identity percentages of the 2nd intron regions were lower than 94% for *Plau*-*S*_2_, *S*_5_, *S*_6_, *S*_7_, *S*_9_, *S*_12_, *S*_15_, *S*_17_, and *S*_19_. This discrepancy is mainly due to the frequency of indels and base substitutions in the coding and non-coding regions of a gene and confirms these *S*-RNases are functional since introns that are under weaker constraints are more rapidly changing over time (Patthy, 2008).

Mutations occurring in the RHV region of the *S-RNase* gene suggest different allele-specificity of the related alleles (Matton et al., 1997; Ushijima et al., 1998). In some cases (e.g., *Plau*-*S*_4_ and *Pvirg*-*S*_2_) it provides further support against the trans-specific origin of the alleles, although mutations may have occurred after speciation, as well. However, in this case the fact that *P. virginiana* also belongs to the Racemose group together with *P. laurocerasus*, the common origin of their alleles should be reflected by a position closer to each other on the phylogenetic tree than to any alleles in species of the Solitary group. All these considerations can help us decide whether the alleles are trans-specific or not. However, the effects of polyploidy should be also considered. *Plau*-*S*_2_ seemed to be trans-specific to both *Pcer*-*S*_35_ and *Pspec*-*S*_34_ but not to *Pav*-*S*_27_. The differences were evident in the amino acid sequences, but introns were even more different. *Pcer*-*S*_35_ is an allele of the tetraploid species, sour cherry, and this allele putatively derived from the ground cherry, another tetraploid species. *P. speciosa* is also tetraploid and *P. laurocerasus* is docosaploid and hence the difference between the alleles of the *P. avium* and the polyploid species might be attributed to the accelerated evolution of paralogous gene copies in polyploid species (Tsukamoto et al., 2006), so their trans-specific origin cannot be ruled out.

The *Plau*-*S*_6_ and *S*_8_ shared considerable similarity and hence their closest homologs found in the database were also identical. They differed in 9 AAs, two of which located in the RHV region. It suggests the two alleles are related but may have different allele-specificity. It should be checked by controlled pollination but *P. laurocerasus* is an extreme polyploid species, and it is hard to predict the effect polyploidy might have on the result of crosses, as it was mentioned by Sutherland et al. (2008) in case of crosses with the hexaploid *P. domestica*.

### The Mutational Frequency in Different Regions of the *S-RNase* Gene

The analysis of closely related *S*-RNase alleles indicated that most of the AA replacements (43%) occurred between the RC4 and C5 regions, following the regions between C3 and RC4 (18%) and RHV (16%). Interestingly, such regions are corresponding to the PS3, PS2, and PS1 regions in *S*-RNases of subtribe Malinae, respectively, where positive selection is likely to operate and contribute to *S*-allele-specificity (Ishimizu et al., 1998).

The RHV was first reported to be responsible for allele-specificity (Ushijima et al., 1998). The function of this region was also confirmed using transgenic *Solanum chacoense* plants (Matton et al., 1997), and it means if alleles had a common origin, the accumulation of amino acid replacements in this region might lead to the change of allele-specificity. It is rather interesting to observe that the regions between C3 and RC4 accumulated even a bit larger number of accepted mutations than RHV. However, the most striking feature in mutational frequency was the 43% of all accepted mutations occurring between RC4 and C5. This region contains an indel with a varying number of AAs ranging from 6 to 25 in the newly identified *P. laurocerasus S*-RNases. This region forms a loop in the 3D protein structure (Fernandez i Marti et al., 2012) that is in general tolerant to indel mutations (Patthy, 2008). The indel events might be frequent in this part of the gene since there is no purifying selection acting against them.

In addition, PS3 (that corresponds to the region between RC4 and C5 in *Prunus S*-RNases) was also a region in the RNases of subtribe Malinae where positive selection is likely, indicating its putative involvement in allele-specificity. It was further supported by Ortega et al. (2006) who reported on six of ten AA differences in the RC4 and C5 regions of almond *S*_11_- and *S*_24_-alleles. The ratio of non-conservative amino acid replacements was the highest in the RHV region (50%), followed by RC4-C5 (33%) and C3-RC4 (28%). Furthermore, in the analysis of 88 *Prunus S*-*RNase* sequences, Vieira et al. (2007) identified 28 amino acid sites under positive selection, most of which (9) occurred in the region RC4-C5, followed by 5 and 4 sites in C3-RC4 and RHV. Our results support that the region just upstream of C5 can have a contribution to allele-specificity of *Prunus S-RNase* gene.

This study provides the first insight into the SI system of cherry laurel by identifying a set of its putative *S-RNase* allele sequences. Our results are based on the specificity of the alleles used and statistically significant homology to other *Prunus S*-alleles. The identified *S*-*RNase* alleles are considered putative because the mechanisms like retroposition or unequal crossing over can result in non-functional paralog gene copies. Although the first can be excluded as our sequences have kept their second intron, experimental confirmation (to check if alleles co-segregate with SI phenotypes) will be required for no-doubt clarification of *S*-RNase function. At least, three non-functional *S*-RNases were also identified that might be useful in further studies to clarify the genetics of self-(in)compatibility in this polyploid *Prunus* species. Our data are consistent with the presumed SI of this species and seem to support the one-allele-match model of transition to SC. The information on the *S-*genotypes may be a key factor in the breeding process of new cherry laurel cultivars suitable for mass fruit production. The availability of cherry laurel *S*-RNase allele sequences may also facilitate the design of allele-specific primers or other assays. Significant similarities and trans-specific origin of the new cherry laurel and other *Prunus S-RNases* have been revealed, which could make the molecular evolutionary analyses more precise, especially by providing data for the less studied Racemose group of *Prunus* phylogeny.

## Data Availability Statement

The datasets presented in this study can be found in online repositories. The names of the repository/repositories and accession number(s) can be found in the article/Supplementary Material.

## Author Contributions

JH and AM performed the molecular analyses and wrote the first draft of the manuscript. GI and SE provided the plant material and information on studied plants. AH performed the bioinformatic studies and wrote parts of the manuscript. All authors contributed to the article and approved the submitted version.

## Conflict of Interest

The authors declare that the research was conducted in the absence of any commercial or financial relationships that could be construed as a potential conflict of interest.

## Publisher’s Note

All claims expressed in this article are solely those of the authors and do not necessarily represent those of their affiliated organizations, or those of the publisher, the editors and the reviewers. Any product that may be evaluated in this article, or claim that may be made by its manufacturer, is not guaranteed or endorsed by the publisher.

## Footnotes

1
http://www.ncbi.nlm.nih.gov

## Supplementary Material

Supplementary Figure 1 Alignment of the genomic DNA sequences of the wild-type and mutated allele pairs of *Prunus laurocerasus S-RNases*. A: *S*_5_ and *S*_5__m_; B: *S*_13_ and *S*_13__m_; and C: *S*_18_ and *S*_18__m_ *RNase* alleles. The 5’ and 3’ ends of the *S-RNase* 2nd intron are indicated by a blue arrow and a blot, respectively. The differences, such as indels, and SNPs are highlighted in black.

Supplementary Table 1 Sizes of the amplification products of PCR analysis conducted using the PaConsI-F (labeled with FAM) and PaConsI-R2 consensus primers, amplifying the first intron of the *Prunus laurocerasus S-RNase* gene. The amplicons were run on an automated capillary sequencer, ABI PRISM 3100 Genetic Analyzer. The ABI Peak Scanner 1.0 software and GS500 LIZ (Applied Biosystems) internal size standard were used for data analysis.

Supplementary Table 2 Percentage (%) of the pairwise identities of the deduced amino acid sequences (upper right half) and nucleotide sequences (lower left half) of *Prunus laurocerasus* partial *S*-*RNase* alleles. The putatively truncated protein sequences of the *S*_5__m_- and *S*_18__m_-alleles were not included in the analysis.

Supplementary Table 3 The results of the BLASTP analysis performed on the deduced amino acid sequence of *Prunus laurocerasus* putative *S-RNase* alleles determined in this work, their corresponding NCBI Protein database accession number, percentage of query coverage and identity, the *E*-value of alignment score and the closest homologs (species name, allele label and accession number).

Supplementary Table 4 Pairwise identity (%) matrix of the deduced amino acid sequences of 20 partial *Prunus laurocerasus* and 58 other *Prunus S*-*RNase* alleles.

Supplementary Table 5 Sequence identity percentages of the 2nd intron and deduced amino acid sequences of *Prunus laurocerasus* and their most similar *Prunus S*-RNases. Green and red background color indicate great and small identities, respectively. Intron identity values are missing when only the mRNA sequence was available and hence, the comparison was not feasible. The asterisk (^∗^) indicates the database sequence was shorter or deficient. Amino acid replacements are shown in different regions of the *S*-RNase protein and amino acid positions. Orange and blue color show mutations occurring in a single sequence or group of sequences, respectively.

## Abbreviations

AA: amino acid
GSI: gametophytic self-incompatibility
SC: self-compatibility
SI: self-incompatibility
*S*-RNase: *S*-ribonuclease.
