# Supplementary material for: Identification and Molecular Analysis of Putative Self-Incompatibility Ribonuclease Alleles in an Extreme Polyploid Species, Prunus laurocerasus L
Source: Front Plant Sci. 2021 Sep 23;12:715414. doi: 10.3389/fpls.2021.715414 (PMC8495262; doi:10.3389/fpls.2021.715414)

**Supplementary Figure S1.** Alignment of the genomic DNA sequences of the wild-type and mutated allele pairs of *Prunus laurocerasus S-RNases*. A: *S*_5_ and *S*_5m_; B: *S*_13_ and *S*_13m_; and C: *S*_18_ and *S*_18m_ *RNase* alleles. The 5’ and 3’ ends of the *S-RNase* 2^nd^ intron are indicated by a blue arrow and a blot, respectively. The differences, such as indels, and SNPs are highlighted in black.

**A**


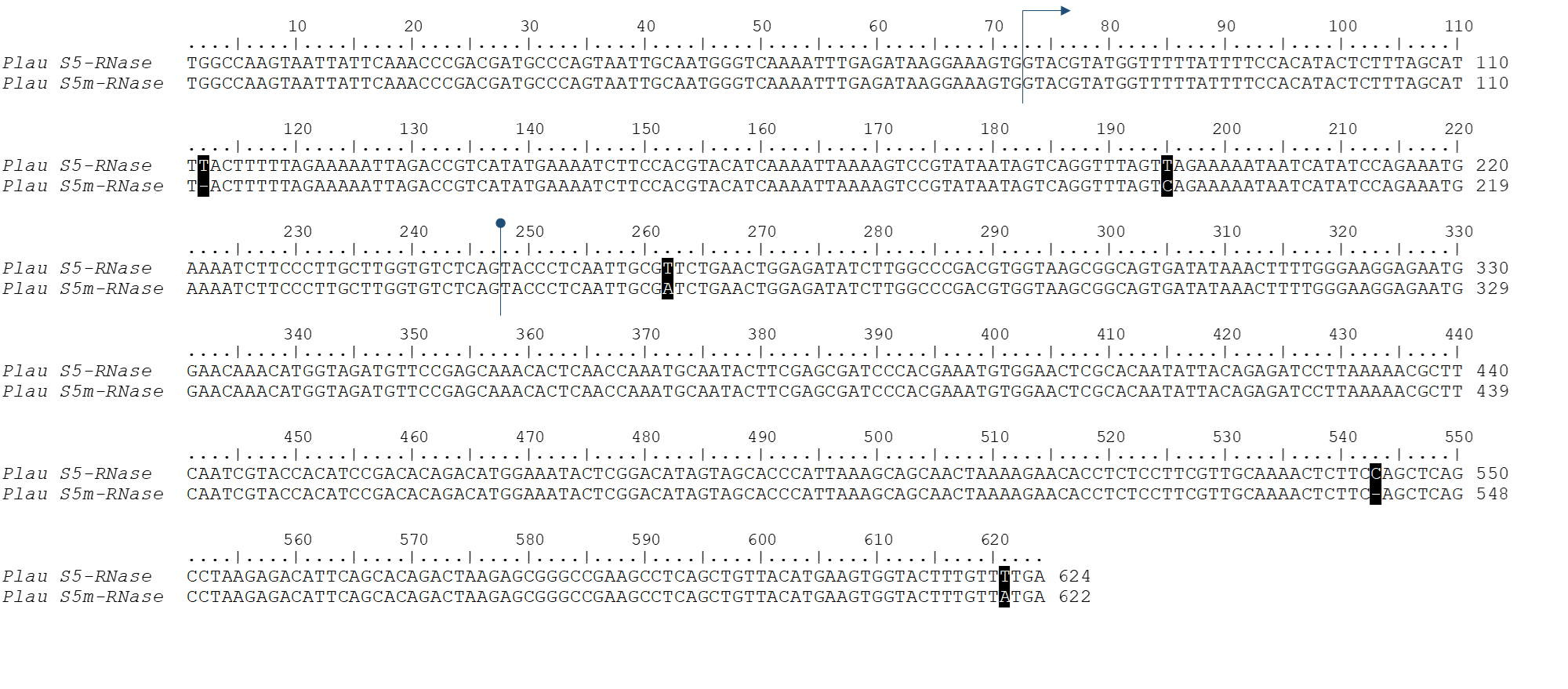


**B**


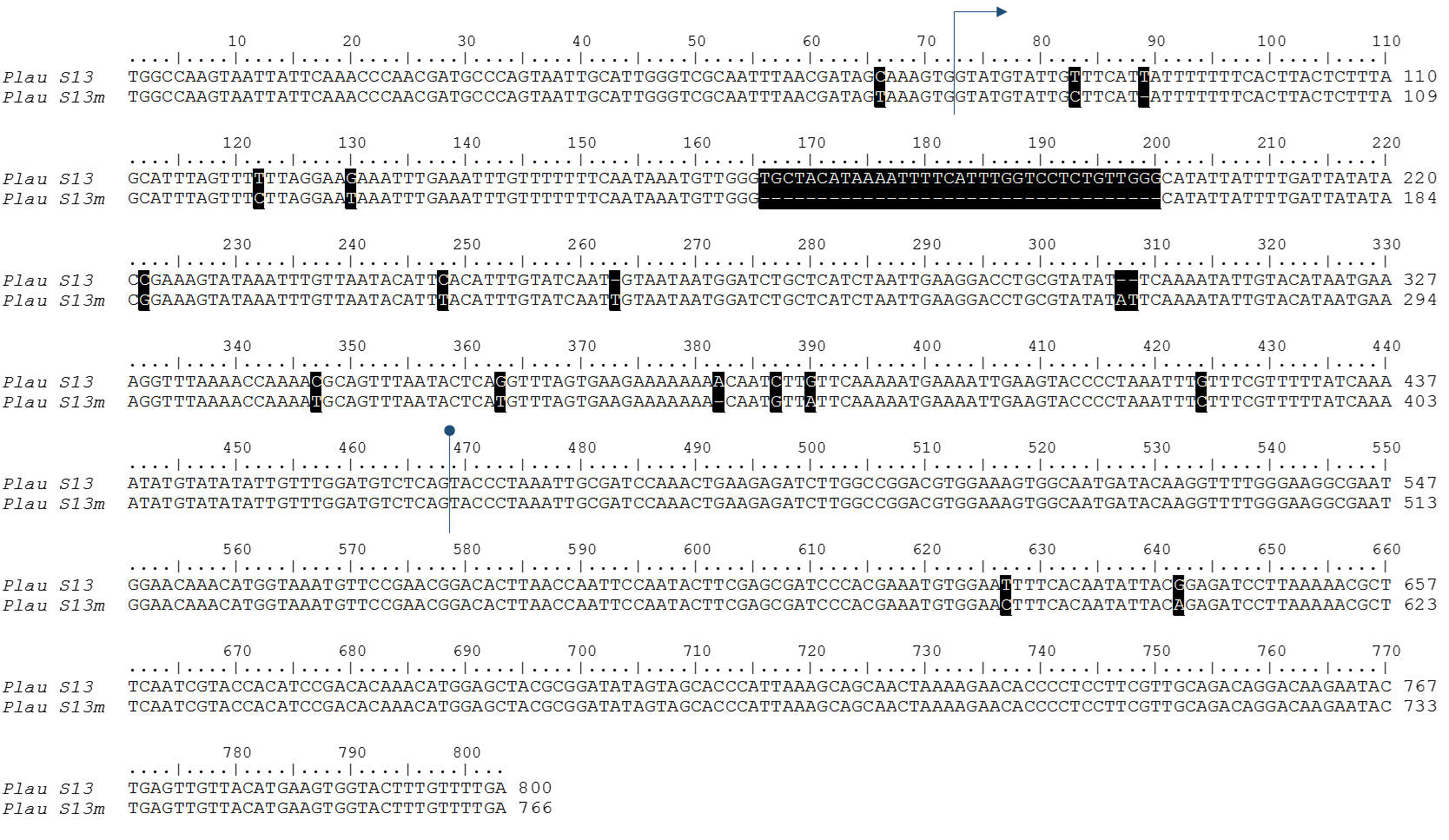


**C**


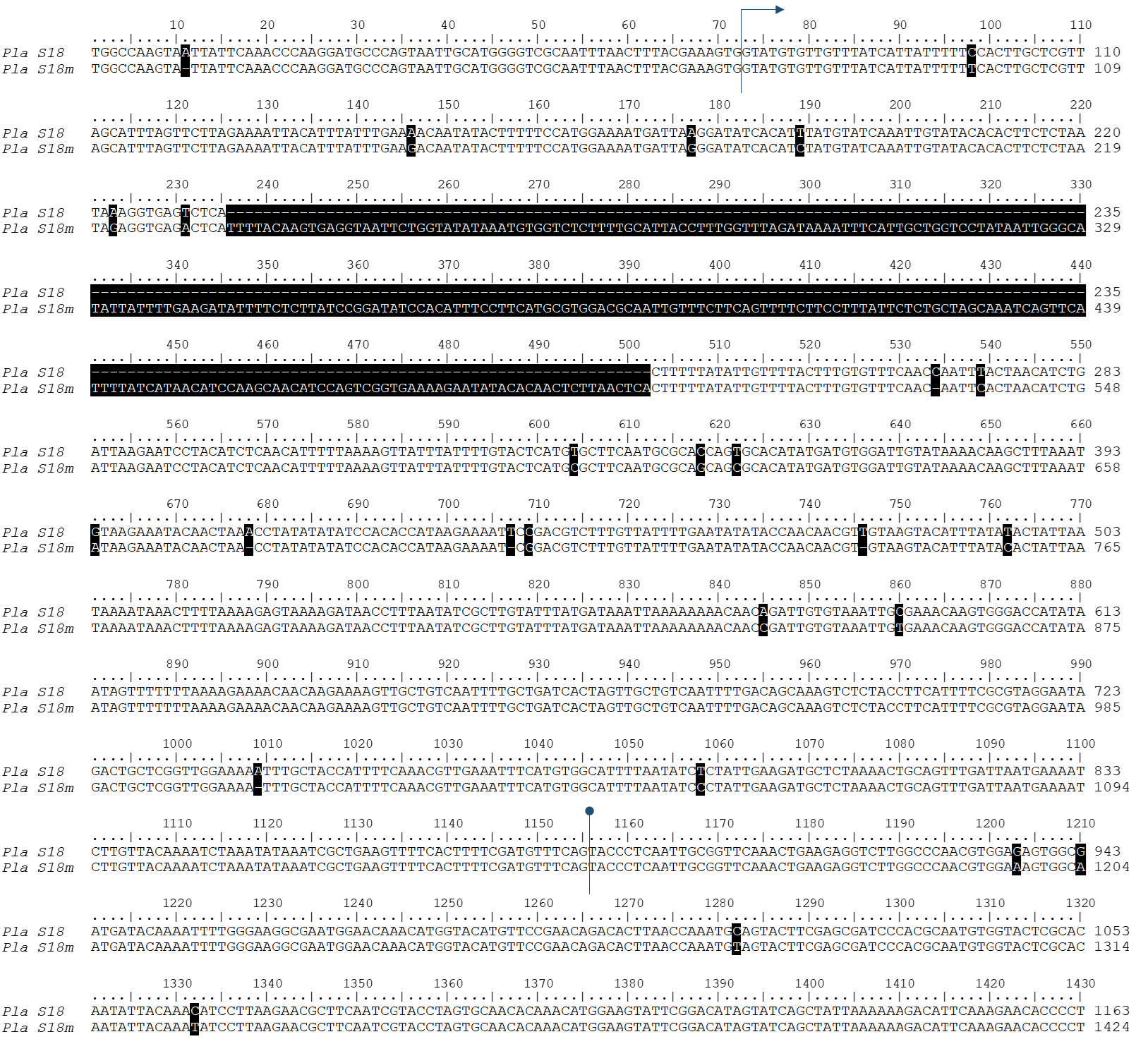

Supplement: Supplementary Figure 1 — Alignment of the genomic DNA sequences of the wild-type and mutated allele pairs of Prunus laurocerasus S-RNases. A: S5 and S5m; B: S13 and S13m; and C: S18 and S18m RNase alleles. The 5’ and 3’ ends of the S-RNase 2nd intron are indicated by a blue arrow and a blot, respectively. The differences, such as indels, and SNPs are highlighted in black. [file Data_Sheet_1.docx]
