# Supplementary material for: Identification and Molecular Analysis of Putative Self-Incompatibility Ribonuclease Alleles in an Extreme Polyploid Species, Prunus laurocerasus L
Source: Front Plant Sci. 2021 Sep 23;12:715414. doi: 10.3389/fpls.2021.715414 (PMC8495262; doi:10.3389/fpls.2021.715414)
Supplement: Supplementary Table 3 — The results of the BLASTP analysis performed on the deduced amino acid sequence of Prunus laurocerasus putative S-RNase alleles determined in this work, their corresponding NCBI Protein database accession number, percentage of query coverage and identity, the E-value of alignment score and the closest homologs (species name, allele label and accession number). [file Table_3.DOCX]

**Supplementary Table S3. |** The results of the BLASTP analysis performed on the deduced amino acid sequence of *Prunus laurocerasus* *S-RNase* alleles determined in this work, their corresponding NCBI Protein database accession number, percentage of query coverage and identity, the *E*-value of alignment score and the closest homologs (species name, allele label and accession number)

| Allele | NCBI Protein accession number | Coverage (%) | Identity (%) | *E*-value | The closest homolog (species and accession number) |
| --- | --- | --- | --- | --- | --- |
| *S*_1_ | [AVI26323.1](https://www.ncbi.nlm.nih.gov/protein/AVI26323.1?report=genbank&log$=prottop&blast_rank=1&RID=7BMXYYCV016) | 100 | 95.74 | 4e-101 | *Prunus speciosa* *S*_22_-*RNase*, ADZ76512 |
|  |  |  |  |  |  |
| *S*_2_ | [AVI26324.1](https://www.ncbi.nlm.nih.gov/protein/AVI26324.1?report=genbank&log$=prottop&blast_rank=1&RID=7BNJJ405016) | 99 | 97.20 | 5e-99 | *Prunus cerasus S35,* ABW74348 |
|  |  | 98 | 95.07 | 5e-96 | *Prunus speciosa* *S*_34_-*RNase*, BAF56271 |
|  |  | 93 | 95.56 | 5e-96 | *P. avium S27,* ABB92549 |
|  |  |  |  |  |  |
| *S*_3_ | [AVI26325.1](https://www.ncbi.nlm.nih.gov/protein/AVI26325.1?report=genbank&log$=prottop&blast_rank=1&RID=7BP2DFKG016) | 100 | 100 | 8e-95 | *Prunus avium S*_6_, ABW71898 |
|  |  | 100 | 97.76 | 4e-93 | *Prunus virginiana S*_4_, AFI24537 |
|  |  | 100 | 97.76 | 1e-92 | *Prunus avium S*_24_, AAP92435 |
|  |  |  |  |  |  |
| *S*_4_ | [AVI26326.1](https://www.ncbi.nlm.nih.gov/protein/AVI26326.1?report=genbank&log$=prottop&blast_rank=1&RID=7BWS3FRY013) | 99 | 99.29 | 1e-100 | *Prunus armeniaca S*_65_, AFH56917 |
|  |  | 100 | 98.59 | 4e-100 | *Prunus armeniaca S*_4_, AAT69248 |
|  |  | 99 | 97.87 | 5e-99 | *Prunus armeniaca S*_48_, AEB96592 |
|  |  | 98 | 99.28 | 1e-98 | *Prunus armeniaca S*_24_, ABS84176 |
|  |  | 98 | 97.86 | 2e-97 | *Prunus armeniaca S*_38_, ADD71779 |
|  |  | 93 | 98.5 | 7e-93 | *Prunus tenella S*_1_, ABL86035 |
|  |  | 97 | 94.24 | 1e-92 | *Prunus virginiana S*_2_*,* AFI24535 |
|  |  | 88 | 98.41 | 1e-87 | *Prunus mira S*_1_, BAK19919 |
|  |  |  |  |  |  |
| *S*_5_ | [AVI26327.1](https://www.ncbi.nlm.nih.gov/protein/AVI26327.1?report=genbank&log$=prottop&blast_rank=1&RID=7BXGJMJ3016) | 98 | 97.28 | 4e-102 | *Prunus virginiana S*_5_, AFJ20685 |
|  |  | 98 | 94.56 | 1e-97 | *Prunus domestica Sf,* QTI57263 |
| *S*_5m_ | MG922592 | 98 | 93.5 | 0.0 | *Prunus virginiana S*_5_, AFJ20685 |
|  |  |  |  |  |  |
| *S*_6_ | [AVI26328.1](https://www.ncbi.nlm.nih.gov/protein/AVI26328.1?report=genbank&log$=prottop&blast_rank=1&RID=7BYZKT8E013) | 100 | 95.56 | 4e-92 | *Prunus mume S*_11_, ABV71999 |
|  |  | 100 | 95.56 | 5e-92 | *Prunus armeniaca S*_17_, ACD31530 |
|  |  | 100 | 94.81 | 1e-91 | *Prunus spinosa S*_3-1_, ABG76209 |
|  |  | 100 | 94.07 | 7e-91 | *Prunus mume S*_15_*,* ABV72003 |
|  |  | 100 | 93.33 | 2e-90 | *Prunus laurocerasus S*_8_, AVE15904 |
|  |  | 98 | 93.23 | 2e-87 | *Prunus virginiana S*_6_*,* AFJ20686 |
| *S*_7_ | [AVI26329.1](https://www.ncbi.nlm.nih.gov/protein/AVI26329.1?report=genbank&log$=prottop&blast_rank=1&RID=7BZZ152E013) | 99 | 95.52 | 4e-92 | *Prunus armeniaca S*_66_, AFH56918 |
|  |  | 98 | 96.24 | 4e-91 | *Prunus dulcis S*_55_, CBI68343 |
|  |  | 98 | 95.49 | 3e-90 | *Prunus armeniaca S*_9_*,* ABO34169 |
|  |  | 98 | 94.74 | 2e-89 | *Prunus dulcis S*_40_*,* AEI69726 |
|  |  | 97 | 96.97 | 1e-88 | *Prunus webbii S*_k_*,* CAM84228 |
| *S*_8_ | [AVE15904.1](https://www.ncbi.nlm.nih.gov/protein/AVE15904.1?report=genbank&log$=prottop&blast_rank=1&RID=7C6C3AHM013) | 100 | 97.04 | 1e-92 | *Prunus spinosa S*_3-1_, ABG76209 |
|  |  | 100 | 96.30 | 3e-92 | *Prunus mume S*_11_*,* ABV71999 |
|  |  | 100 | 96.30 | 5e-92 | *Prunus armeniaca S*_17_*,* ACD31530 |
|  |  | 100 | 94.81 | 6e-91 | *Prunus mume S*_15_*,* ABV72003 |
|  |  | 100 | 93.33 | 2e-90 | *Prunus laurocerasus S*_6_*,* AVI26328 |
|  |  | 100 | 93.98 | 6e-88 | *Prunus virginiana S*_6_*,* AFJ20686 |
| *S*_9_ | [AVE15905.1](https://www.ncbi.nlm.nih.gov/protein/AVE15905.1?report=genbank&log$=prottop&blast_rank=1&RID=7C7C2GWU013) | 100 | 99.27 | 3e-98 | *Prunus avium S*_13_, ABD49101 |
|  |  | 100 | 98.54 | 6e-97 | *Prunus tenella S*_17_*,* AMY98982 |
| *S*_10_ | [AZP02726.1](https://www.ncbi.nlm.nih.gov/protein/AZP02726.1?report=genbank&log$=prottop&blast_rank=1&RID=7C7PAFJG013) | 98 | 85.5 | 0.0 | *Prunus dulcis S*_54_, AY613341 |
|  |  | 99 | 93.24 | 3e-97 | *Prunus armeniaca S*_50_*,* AEB96594 |
|  |  | 98 | 95.24 | 1e-96 | *Prunus virginiana S*_7_*,* AFJ20687 |
|  |  | 97 | 94.52 | 3e-96 | *Prunus armeniaca S*_49_*,* AEB96593 |
|  |  | 97 | 93.84 | 4e-95 | *Prunus dulcis S*_63_*,* AAT72309 |
| *S*_11_ | [AZP02727.1](https://www.ncbi.nlm.nih.gov/protein/AZP02727.1?report=genbank&log$=prottop&blast_rank=1&RID=7C87G2TY016) | 98 | 97.76 | 6e-93 | *Prunus speciosa S*_3_, BAF56244 |
|  |  | 98 | 96.27 | 5e-90 | *Prunus virginiana S*_6_*,* AFI24539 |
|  |  | 93 | 98.43 | 2e-89 | *Prunus armeniaca S*_40_, ADC97909 |
|  |  |  |  |  |  |
| *S*_12_ | [AZP02728.1](https://www.ncbi.nlm.nih.gov/protein/AZP02728.1?report=genbank&log$=prottop&blast_rank=1&RID=7C8P26MJ013) | 100 | 97.89 | 7e-99 | *Prunus salicina S*_h_, ABF61823 |
|  |  | 98 | 98.57 | 2e-97 | *Prunus simonii S*_1_, ACG50928 |
|  |  | 98 | 95.71 | 1e-94 | *Prunus speciosa S*_44_, BAF56279 |
|  |  |  |  |  |  |
| *S*_13_ | [AZP02729.1](https://www.ncbi.nlm.nih.gov/protein/AZP02729.1?report=genbank&log$=prottop&blast_rank=1&RID=7C9CJFHH016) | 87 | 100 | 2e-83 | *Prunus avium S*_9_, CAG25687 |
|  |  |  |  |  |  |
| *S*_13m_ | AZP02730 |  |  |  | *Prunus avium S*_9_, CAG25687 |
|  |  |  |  |  |  |
| *S*_14_ | [AZP02731.1](https://www.ncbi.nlm.nih.gov/protein/AZP02731.1?report=genbank&log$=prottop&blast_rank=1&RID=7C9P4RP3013) | 100 | 90.37 | 1e-87 | *Prunus dulcis S*_9_, ATQ63985 |
|  |  |  |  |  |  |
| *S*_15_ | [AZP02732.1](https://www.ncbi.nlm.nih.gov/protein/AZP02732.1?report=genbank&log$=prottop&blast_rank=1&RID=7CA6E17R013) | 100 | 95.65 | 1e-94 | *Prunus spinosa S*_7_, ACJ22520 |
|  |  |  |  |  |  |
| *S*_16_ | [AZP02733.1](https://www.ncbi.nlm.nih.gov/protein/AZP02733.1?report=genbank&log$=prottop&blast_rank=1&RID=7CADAKRZ013) | 70 | 93.4 | 2e-163 | *Prunus japonica S*_1_, EF635417 |
|  |  |  |  |  |  |
| *S*_17_ | [AZP02734.1](https://www.ncbi.nlm.nih.gov/protein/AZP02734.1?report=genbank&log$=prottop&blast_rank=1&RID=7CAJDVDH013) | 97 | 97.84 | 3e-95 | *Prunus tenella S*_13_, AJP09160 |
|  |  | 83 | 99.15 | 1e-81 | *Prunus serotina S*_3_, QHZ99306 |
| *S*_18_ | [AZP02735.1](https://www.ncbi.nlm.nih.gov/protein/AZP02735.1?report=genbank&log$=prottop&blast_rank=1&RID=7CAYB8HR013) | 100 | 86.96 | 4e-83 | *Prunus armeniaca S*_C_, ABE27180 |
| *S*_18m_ | MG922593 |  |  |  |  |
|  |  |  |  |  |  |
| *S*_19_ | [AZP02736.1](https://www.ncbi.nlm.nih.gov/protein/AZP02736.1?report=genbank&log$=prottop&blast_rank=1&RID=7CB536WX013) | 97 | 99.26 | 1e-95 | *Prunus armeniaca S*_25_, ABS84177 |
|  |  | 100 | 96.40 | 9e-95 | *Prunus tenella S*_9_, [AAZ06135.1](https://www.ncbi.nlm.nih.gov/protein/AAZ06135.1?report=genbank&log$=prottop&blast_rank=3&RID=8XUFVYVS013) |
|  |  | 100 | 96.40 | 8e-95 | *Prunus dulcis S*_n_, AAZ06135 |
|  |  | 98 | 97.08 | 2e-93 | *Prunus dulcis S*_54_, CBI68342 |
|  |  |  |  |  |  |
| *S*_20_ | [AZP02737.1](https://www.ncbi.nlm.nih.gov/protein/AZP02737.1?report=genbank&log$=prottop&blast_rank=1&RID=7CBRH8SF016) | 100 | 80.99 | 6e-79 | *Prunus armeniaca S*_1_, AAT69244 |
